# Association Between Laparoscopically Confirmed Endometriosis and Risk of Early Natural Menopause

**DOI:** 10.1001/jamanetworkopen.2021.44391

**Published:** 2022-01-21

**Authors:** Madhavi Thombre Kulkarni, Amy Shafrir, Leslie V. Farland, Kathryn L. Terry, Brian W. Whitcomb, A. Heather Eliassen, Elizabeth R. Bertone-Johnson, Stacey A. Missmer

**Affiliations:** 1 Department of Obstetrics, Gynecology, Reproductive Biology, College of Human Medicine, Michigan State University, East Lansing; 2 Division of Adolescent and Young Adult Medicine, Department of Medicine, Boston Children's Hospital and Harvard Medical School, Boston, Massachusetts; 3 Department of Epidemiology and Biostatistics, Mel and Enid Zuckerman College of Public Health, Tucson, Arizona; 4 Department of Obstetrics and Gynecology, College of Medicine–Tucson, University of Arizona, Tucson; 5 Obstetrics, Gynecology Epidemiology Center, Department of Obstetrics and Gynecology, Brigham and Women’s Hospital and Harvard Medical School, Boston, Massachusetts; 6 Department of Epidemiology, Harvard T.H. Chan School of Public Health, Boston, Massachusetts; 7 Department of Biostatistics and Epidemiology, School of Public Health and Health Sciences, University of Massachusetts, Amherst; 8 Channing Division of Network Medicine, Department of Medicine, Brigham and Women’s Hospital and Harvard Medical School, Boston, Massachusetts; 9 Department of Health Promotion and Policy, School of Public Health and Health Sciences, University of Massachusetts, Amherst; 10 Department of Obstetrics, Gynecology, and Reproductive Biology, Michigan State University, Grand Rapids

## Abstract

**Importance:**

Early natural menopause (ENM) has been associated with reduced reproductive span, cardiovascular disease risk, and early mortality. The potential adverse implications of endometrioma surgery for ovarian reserve are known, yet the association of endometriosis with menopausal timing remains understudied.

**Objective:**

To investigate the association between endometriosis and risk for ENM.

**Design, Setting, and Participants:**

This large, population-based cohort study analyzed data from the Nurses’ Health Study II cohort questionnaires from the 1989 to 2015 questionnaire cycles. The sample included premenopausal women aged 25 to 42 years at baseline or enrollment in 1989. Cumulative follow-up rate was greater than 90%, and participants continued follow-up until the onset of ENM, age 45 years, hysterectomy, oophorectomy, cancer diagnosis, death, loss to follow-up, or end of follow-up in May 2017, whichever occurred first. Data analyses were conducted from October 26, 2020, to April 27, 2021.

**Exposures:**

Endometriosis diagnosis status was queried in the biennial questionnaires, with participants reporting physician diagnosis and whether the diagnosis was laparoscopically confirmed.

**Main Outcomes and Measures:**

Natural menopause before age 45 years. Menopause status was assessed every 2 years.

**Results:**

The study included 106 633 premenopausal women with a mean (SD) age of 34.8 (4.3) years at baseline, of whom 3921 reported a laparoscopically confirmed endometriosis diagnosis. During 1 508 462 person-years of follow-up, 6640 participants reported being diagnosed with endometriosis, 99 993 never reported endometriosis, and 2542 reported experiencing ENM. In the age- and calendar time–adjusted model, laparoscopically confirmed endometriosis was associated with a 50% greater risk for ENM (hazard ratio [HR], 1.51; 95% CI, 1.30-1.74). A similar risk was observed after adjusting for race and ethnicity and time-varying anthropometric and behavioral factors (HR, 1.46; 95% CI, 1.26-1.69). With additional adjustment for reproductive factors, the HR of ENM was attenuated but significant (HR, 1.28; 95% CI, 1.10-1.48). A greater risk of ENM was observed among women who were nulliparous after stratifying by parity (nulliparous vs parous: HR, 1.46 [95% CI, 1.15-1.86] vs 1.14 [95% CI, 0.94-1.39]; *P* for heterogeneity = .05) or who never used oral contraceptives when stratifying by oral contraceptive use (never vs ever: HR, 2.03 [95% CI, 1.34-3.06] vs 1.20 [95% CI, 1.02-1.42]; *P* for heterogeneity = .02). No significant differences were observed in the association between endometriosis and ENM when stratifying by body mass index (calculated as weight in kilograms divided by height in meters squared; <25 vs ≥25: HR, 1.20 [95% CI, 0.99-1.45] vs 1.43 [95% CI, 1.11-1.83; *P* for heterogeneity = .34), cigarette smoking status (never vs ever: HR, 1.36 [95% CI, 1.13-1.65] vs 1.11 [95% CI, 0.87-1.42]; *P* for heterogeneity = .57), or history of infertility attributed to ovulatory disorder (no vs yes: HR, 1.28 [95% CI, 1.08-1.51] vs 1.28 [95% CI, 0.90-1.82]; *P* for heterogeneity = .86).

**Conclusions and Relevance:**

This cohort study found a risk for ENM in women with laparoscopically confirmed endometriosis. These women compared with those without endometriosis may be at a higher risk for shortened reproductive duration, particularly those who were nulliparous or never used oral contraceptives.

## Key Points

**Question:**

Is there an association between endometriosis and early natural menopause?

**Findings:**

In this cohort study of 106 633 premenopausal women, a statistically significant association was found between endometriosis and early natural menopause (before age 45 years) after accounting for demographic, behavioral, and reproductive factors. The risk was greater for nulliparous women and women who never reported using oral contraceptives.

**Meaning:**

The findings of this study suggest that women with endometriosis may be at a higher risk for a shortened reproductive duration.

## Teaser

This cohort study investigates the association of endometriosis and risk of early menopause, adjusting for assesses the anthropometric, behavioral, and reproductive factors, among premenopausal female nurses in the Nurses’ Health Study II cohort.

## Introduction

Endometriosis is an often chronic inflammatory disease characterized by the presence of endometrial-like tissue outside of the uterus.^1,2^ Although endometriosis affects approximately 10% of women during their reproductive years,^3,4^ its association with other health outcomes, including early natural menopause (ENM), remain unclear.^5,6,7^ Early menopause is defined as the cessation of ovarian function before age 45 years. Affecting approximately 10% of women in Western populations,^8^ endometriosis is associated with greater risk for cardiovascular disease, cognitive decline, osteoporosis, and premature mortality.^9,10^ Multiple mechanisms support the hypothesis that endometriosis or, specifically, endometriomas (the endometriosis subtype characterized by ovarian cysts)^2^ and the treatments associated with it may have qualitative and quantitative implications for ovarian reserve.^11^ Endometriosis has been associated with lower ovarian reserve among women with female-factor infertility,^12,13^ and endometriomas^14^ have been associated with ovarian aging and menopause timing.^11^ In addition, local and systemic increased inflammation has been associated with endometriosis^1,2^ and hypothesized to be associated with early menopause; however, these associations were nonlinear and inconsistent among markers.^15^ Women with endometriosis compared with women without endometriosis have peritoneal fluid that contains more activated proinflammatory, chemotactic, and oxidative stress factors,^16,17^ which could create an environment that is detrimental to follicular and ovarian function and leads to an earlier age at menopause.^18^

Determinants of age at menopause include the number of oocytes at birth, the rate of atresia throughout the lifespan, and the threshold number of oocytes needed to produce sufficient hormones to maintain menstrual cyclicity.^8,19,20^ Reproductive events that interrupt ovulation (eg, pregnancies and lactation)^21^ or modify the rate of atresia slow the depletion of the ovarian follicle pool and are associated with delayed menopause.^21,22^ Conversely, events that disrupt ovarian function, such as ovarian surgery, autoimmune oophoritis, cigarette smoking, and androgenizing exogenous hormones,^22^ may have implications for ovarian reserve, characterized by decreased follicle numbers and lower oocyte quality and quantity, which are associated with earlier age at menopause.^8^ Early natural menopause and endometriosis share several risk factors, including body size,^23,24^ earlier age at menarche,^24^ shorter menstrual cycles,^24,25^ nulliparity,^4,21^ and infertility.^4^ Furthermore, medical and surgical management for endometriosis, including oral contraceptives (OCs)^26^ and ovarian surgeries,^14^ may alter early menopause risk.

Within the UK Royal College of General Practitioners' Oral Contraception Study cohort,^27^ history of endometriosis was associated with ENM. However, the Royal College of General Practitioners' Oral Contraception Study was limited by its reliance on recalled age at menopause and minimal adjustment for potential confounders. Therefore, in the current prospective cohort study, we sought to investigate the association between endometriosis and the risk of ENM using the US Nurses’ Health Study II cohort. We applied a prospective time-to-event analysis that accounted for time-varying anthropometric, behavioral, and reproductive factors.^28^ In addition, we investigated whether the association between endometriosis and ENM risk differed by the following risk factors of age at menopause: body mass index (BMI; calculated as weight in kilograms divided by height in meters squared), cigarette smoking, OC use, parity, and history of infertility that was attributed to ovulatory disorder.

## Methods

The protocol for this cohort study was approved by the institutional review boards at Brigham and Women’s Hospital and Harvard T.H. Chan School of Public Health, which approved that the participants’ ongoing completion of questionnaires would be considered as implied consent. We followed the Strengthening the Reporting of Observational Studies in Epidemiology (STROBE) reporting guideline.

### Study Population

The Nurses’ Health Study II has an ongoing cohort of 116 429 female registered nurses who were aged 25 to 42 years when they responded to the baseline questionnaire in 1989. Participants provided information on behavioral and health-related factors. Since the baseline questionnaire, participants have completed biennial questionnaires to report new diagnoses of medical conditions and to provide updates on dynamic personal characteristics and behaviors. Questionnaire response rates have been high, with a cumulative follow-up rate higher than 90%.

The current study used data from the 1989 to 2015 questionnaires. We limited inclusion eligibility to participants who reported being premenopausal and having no previous cancer diagnosis (except nonmelanoma skin cancer), hysterectomy, or oophorectomy at baseline. Women who were not premenopausal (n = 7638), reported cancer (n = 692), had undergone hysterectomy or oophorectomy (n = 185), or did not participate beyond 1989 (n = 1281) were excluded. A total of 106 633 premenopausal women remained for analyses, who were followed up from 1989 until the return of the 2015 questionnaire (which was closed to data collection on May 31, 2017).

### Assessment of Endometriosis

Participants were asked on each biennial questionnaire from 1993 onward whether they had physician-diagnosed endometriosis. Those who responded yes indicated the year of diagnosis and whether it had been confirmed by laparoscopy, the clinical criterion standard.^2^ Among women with self-reported laparoscopic confirmation of their endometriosis diagnosis, medical record confirmation was high in 2 validation studies (100% in the first wave and 95% in the second wave of the study).^29^ Endometriosis diagnosis status was treated as a time-varying factor and updated over time. Once a participant reported laparoscopically confirmed endometriosis, she was considered to have endometriosis (contributing person-time to the endometriosis-exposed group) through the remainder of the follow-up period.

Participants who reported physician-diagnosed endometriosis but not surgically confirmed endometriosis throughout the follow-up period did not contribute person-time to either the exposed or the unexposed group, as it could not be definitively ascertained that they had endometriosis or confirmed that they did not.^29^ If laparoscopic confirmation was reported at any time after the initial report of physician-only diagnosis, the person-time contribution to the endometriosis-exposed group was reset to the time of the first report because the surgical confirmation validated the initial physician diagnosis.^29^ At baseline in 1989, there were 3921 women who had a history of endometriosis diagnosis, whereas 6640 women were diagnosed with endometriosis after Nurses’ Health Study II enrollment.

### Assessment of Early Menopause

On the 1989 and subsequent biennial questionnaires, participants were asked if their menstruation had ceased permanently. Those who reported that their menstruation had ceased were asked to indicate the age at cessation and whether cessation was related to surgery, radiotherapy, or chemotherapy or occurred naturally. Age at menopause was defined as age after 12 consecutive months of amenorrhea. For women who reported being postmenopausal on 1 questionnaire and then subsequently reported being premenopausal, we defined age at menopause as the age after which menses were absent for 12 months or more and then confirmed that this status persisted for at least 3 consecutive questionnaires. Information regarding cessation was provided on each questionnaire, which enabled us to account for transient cycles without menses followed by the return of menstruation and hence to avoid the misclassification of early menopause status. We defined cases of ENM as women who reported natural menopause before the age of 45 years.

### Covariates

Factors associated with endometriosis or ENM were identified from previous literature as a priori potential confounders^30^ of this hypothesized association. The baseline questionnaire in 1989 collected static information, including birthdate (which was used to calculate current age at enrollment and at all subsequent questionnaire returns), height, race and ethnicity, age at menarche, and menstrual cycle length and regularity during high school and college years. Race and ethnicity data were self-reported by participants at baseline and in 2005, and the racial and ethnic categories followed federal standards. Most of the participants in the cohort identified as White individuals, and thus those in the other racial and ethnic groups (American Indian or Native American, Asian, Black, Hawaiian, multiracial, and other) were combined to form the other category.

All questionnaires collected information to update dynamic factors, such as weight, cigarette smoking status, OC use and duration, parity (OC use and parity were last updated on the 2009 questionnaire, which closed to data collection in 2011 when the youngest participants were aged 47 years), lactation duration, and history of infertility. Participants were asked if they ever tried to become pregnant for more than 1 year without success, if their infertility was clinically evaluated, and if the cause of their infertility was identified. Baseline height and updated weight measurements were used to calculate BMI for each questionnaire cycle. Physical activity was assessed in 1989, 1991, 1997, 2001, 2005, 2009, and 2013, when participants were asked to state the average amount of time they spent each week performing a variety of recreational physical activities. This information was used to calculate metabolic equivalent task hours per week.^30^

Semiquantitative food frequency questionnaires were completed every 4 years starting in 1991 and ending in 2015. The questionnaires assessed the intake of 131 foods and beverages^30,31^ and asked participants to estimate how often they consumed specific items during the preceding year. Vegetable protein intake and alcohol consumption were calculated, and these measures were adjusted for energy using the residual method.^30^ Predicted plasma 25-hydroxyvitamin D (25[OH]D) score was computed using validated statistical models that included major factors of vitamin D status (age, season of blood draw, race and ethnicity, UV-B flux, physical activity, BMI, dietary and supplementary vitamin D intake, and alcohol consumption).^32^

Following are all of the covariates that were used in multivariable models: model 1 was adjusted for age and calendar time; model 2 included model 1 and was additionally adjusted for race and ethnicity (non-Hispanic White or other), BMI at 18 years (<18.5, 18.5 to <22.5, 22.5 to <25, or ≥25), current BMI (<18.5, 18.5 to <22.5, 22.5 to <25, 25 to <30, or ≥30), cigarette smoking status (never, past, or current), cigarette smoking pack-years (0, 1-5, 6-10, 11-15, 16-20, or 21-98), alcohol use (0, 0.1 to <10.0, 10 to 30, or ≥30 g/d), physical activity (<3, 3 to 8, 9 to 17, 18 to 26, or ≥27 metabolic equivalent task hours per week), vegetable protein (quintiles), and estimated plasma 25(OH)D score (ng/mL [to convert to nmol/L, multiply by 2.496]; quintiles); and model 3 included model 2 and was additionally adjusted for age at menarche (≤11, 12-13, or ≥14 years), menstrual cycle length at age 18 to 22 years (<26, 26-31, or ≥32 days), OC use (never, past, or current), duration of OC use (0, 1-23, 24-47, 48-71, 72-95, 96-119, or ≥120 months), parity (nulliparous or parous: 1, 2, 3, or ≥4 children), total lactation duration (0 to <1, 1-6, >6-12, or ≥12 months), and history of infertility attributed to ovulatory disorder (no or yes). Most variables had minimal missing values, with only 3 variables having greater than 0.05% (alcohol use, 6.5%; vegetable protein, 5.1%; and 25[OH]D, 16.6%) missing values.

### Statistical Analysis

Participants contributed person-months to the Nurses’ Health Study II cohort from the return of the baseline questionnaire until (1) the onset of menopause; (2) 45 years of age; (3) a hysterectomy; (4) a bilateral or unilateral oophorectomy; (5) a cancer diagnosis, not including nonmelanoma skin cancer; (6) death; (7) loss to follow-up; or (8) the end of follow-up (May 2017), whichever occurred first. Baseline characteristics of the participants were examined according to baseline endometriosis diagnosis status: laparoscopically confirmed endometriosis (with or without). We used a Cox proportional hazards regression model stratified by age and questionnaire cycle to estimate age- and calendar time–adjusted hazard ratios (HRs) and 95% CIs of the association between laparoscopically confirmed endometriosis and ENM. Proportional hazards assumptions were tested using the likelihood ratio test, which compared models with and without an interaction term for calendar time. In addition to adjusting for age and calendar time, based on previous literature, we adjusted for known anthropometric, demographic, and behavioral potential confounders for endometriosis and ENM a priori, with time-varying covariates updated biennially at every questionnaire cycle. Multivariable model 3 was also adjusted for reproductive risk factors for both endometriosis and ENM. In all multivariable models, we applied the missing indicator method in which an indicator variable was created to address missing values of covariates.^5,33^

Stratified analyses assessed heterogeneity by potential effect modifiers, including BMI (<25 or ≥25), cigarette smoking status (never or ever), OC use (never or ever), parity (nulliparous or parous), and history of infertility attributed to ovulatory disorder (no or yes). Likelihood ratio tests were used to identify statistically significant differences between potential effect modifiers on the multiplicative scale.

We conducted several secondary analyses. To evaluate whether hormone use affected recognition and reporting of menopause, we conducted analyses that included censoring at the first report of hormone therapy (HT) and controlling for HT in multivariable models. Oral contraceptive use can also mask menopause; thus, we conducted analyses that censored at the first report of OC use. Given the conflicting evidence in the literature about the association of OC use with ENM, we also performed an analysis that excluded OC use as a confounder. Given the inconsistent evidence in the literature about the association between inflammatory markers and ENM and given that anti-inflammatory medications were more likely to be used by women with endometriosis, we evaluated potential confounding and mediation effects of nonsteroidal anti-inflammatory drugs. We applied the Cox proportional hazards regression model with and without adjustment for years of use of aspirin, acetaminophen, ibugesic, nonsteroidal anti-inflammatory drugs, and cyclooxygenase inhibitors (modeled continuously). Mediation by medication use was assessed with the difference method,^34^ which was calculated by the percentage change in the β coefficients between the 2 models as follows: (unadjusted β − unadjusted β) divided by unadjusted β.

Clean and complete data for this analysis were available in February 2020. Data analyses were conducted from October 26, 2020, to April 27, 2021. Hypothesis tests were 2-sided for all analyses. *P* ≤ .05 indicated statistical significance. All analyses were performed using SAS, version 9.4 (SAS Institute Inc).

## Results

The characteristics at baseline of the 106 633 female participants (mean [SD] age, 34.8 [4.3] years) are shown in Table 1. A total of 3921 participants reported a history of laparoscopically confirmed endometriosis (3.7% of the eligible participants), and 102 712 participants reported no history of endometriosis. At baseline, small differences by endometriosis status were observed for age, cigarette smoking, alcohol use, physical activity, vegetable protein intake (reported in 1991), estimated plasma 25(OH)D score, and race and ethnicity (Table 1). A larger proportion of women with laparoscopically confirmed endometriosis had a BMI at age 18 years lower than 18.5 compared with women without endometriosis (19.8% vs 14.5%). Women with endometriosis vs those without endometriosis were less likely to report that they had never used OCs (11.3% vs 17.3%) and were more likely to report current use of acetaminophen (26.4% vs 21.3%) or nonsteroidal anti-inflammatory drugs (25.2% vs 18.6%), history of infertility (58.6% vs 16.1%), infertility attributed to ovulatory disorder (15.4% vs 4.3%), and nulliparity (43.6% vs 30.4%) (Table 1).

**Table 1.** Age-Standardized Characteristics of the Study Population at Baseline by Laparoscopically Confirmed Endometriosis Diagnosis

Characteristic^a^	Participants by laparoscopically confirmed endometriosis status, No. (%)	
	With (n = 3921)	Without (n = 102 712)
Age, mean (SD), y^b^	35.2 (4.1)	34.5 (4.6)
Pack-years of cigarette smoking, mean (SD)	4.0 (7.3)	3.8 (7.0)
Alcohol use, mean (SD), g/d	2.9 (4.9)	3.0 (5.7)
Physical activity, MET h/wk, mean (SD)	27.5 (66.2)	28.5 (69.6)
Vegetable protein (% energy), mean (SD)	4.4 (2.9)	4.5 (2.9)
Estimated plasma 25(OH)D score, mean (SD), ng/mL	31.5 (3.4)	31.0 (3.7)
Parity, No. of pregnancies ≥6 mo, mean (SD)	1.1 (1.1)	1.5 (1.2)
Lactation duration, mean (SD), mo^c^	11.2 (11.3)	13.2 (13.2)
Race and ethnicity^d^		
Non-Hispanic White	3802 (97.0)	98 230 (95.6)
Other^e^	119 (3.0)	4482 (4.4)
BMI at age 18 y		
<18.5	771 (19.8)	14 784 (14.5)
18.5 to <22.5	2363 (60.7)	61 807 (60.8)
22.5 to <25	479 (12.3)	14 443 (14.2)
≥25	280 (7.2)	10 611 (10.4)
BMI at baseline		
<18.5	160 (4.1)	3495 (3.4)
18.5 to <22.5	1838 (47.2)	45 534 (44.6)
22.5 to <25	883 (22.7)	22 793 (22.3)
25 to <30	710 (18.2)	18 775 (18.4)
≥30	303 (7.8)	11 396 (11.2)
Cigarette smoking status		
Never	2550 (65.1)	67 478 (65.8)
Past	860 (22.0)	21 802 (21.3)
Current	506 (12.9)	13 284 (13.0)
Aspirin use		
Nonuser	3476 (88.6)	91 699 (89.3)
Current user, <5 y	445 (11.4)	11 013 (10.7)
Acetaminophen use		
Nonuser	2887 (73.6)	80 790 (78.7)
Current user, <5 y	1034 (26.4)	21 922 (21.3)
NSAID use		
Nonuser	2932 (74.8)	83 609 (81.4)
Current user, <5 y	989 (25.2)	19 103 (18.6)
Age at menarche, y		
<11	1049 (26.7)	24 723 (24.1)
12-13	2251 (57.4)	59 455 (57.9)
≥14	621 (15.8)	18 534 (18.0)
Menstrual cycle length at age 18-22 y, d		
<26	439 (11.3)	11 761 (11.5)
26-31	2664 (68.3)	67 751 (66.1)
≥32	799 (20.5)	22 925 (22.4)
Menstrual cycle pattern at age 18-22 y		
Regular	2862 (75.1)	76 223 (76.5)
Irregular	946 (24.8)	23 187 (23.3)
No menstruation	5 (0.1)	264 (0.3)
OC use		
Never	445 (11.3)	17 719 (17.3)
Past	3046 (77.7)	72 336 (70.5)
Current	429 (11.0)	12 526 (12.2)
Duration of OC use, mo		
0	445 (11.5)	17 719 (17.6)
1-23	940 (24.4)	21 632 (21.5)
24-47	855 (22.2)	19 747 (19.6)
48-119	1324 (34.3)	33 122 (32.9)
≥120	295 (7.7)	8528 (8.5)
Infertility history		
Yes	2298 (58.6)	16 500 (16.1)
No	1623 (41.4)	86 212 (83.9)
Infertility cause		
Attributed to ovulatory disorder	605 (15.4)	4388 (4.3)
Not attributed to ovulatory disorder	1694 (43.2)	12 112 (11.8)
Nulliparous		
Yes	1708 (43.6)	31 213 (30.4)
No	2213 (56.4)	71 480 (69.6)

Abbreviations: 25(OH)D, 25-hydroxyvitamin D; BMI, body mass index (calculated as weight in kilograms divided by height in meters squared); MET, metabolic equivalent task; NSAID, nonsteroidal anti-inflammatory drug; OC, oral contraceptive.

SI conversion factor: To convert estimated plasma 25(OH)D to nanomoles per liter, multiply by 2.496.

^a^ Values of categorical variables may not sum to 100% because of rounding.

^b^ Value is not age adjusted.

^c^ Among parous women only.

^d^ Race and ethnicity data were self-reported by participants at baseline and in 2005, and racial and ethnic categories followed federal standards. Most of the participants in the cohort identified as White individuals, and thus those in the other racial and ethnic groups were combined to form the other category.

^e^ Other category included individuals identifiying as American Indian or Native American, Asian, Black, Hawaiian, multiracial, and other.

Tests of proportionality of hazards did not suggest a violation of assumptions. After 1 508 462 person-years of follow-up, 6640 participants reported being diagnosed with endometriosis, 99 993 reported having no endometriosis diagnosis, and 2542 reported experiencing ENM only. In the age- and calendar time–adjusted model (model 1), women with laparoscopically confirmed endometriosis had 50% greater subsequent risk of ENM (HR, 1.51; 95% CI, 1.30-1.74) compared with women without physician-diagnosed endometriosis (Table 2). A similar magnitude of risk was observed under model 2 after adjusting for race and ethnicity and time-varying BMI and behavioral factors (HR, 1.46; 95% CI, 1.26-1.69). The HR was attenuated to a 30% greater risk of ENM but remained statistically significant under model 3, with additional adjustments for reproductive factors (HR, 1.28; 95% CI, 1.10-1.48) (Table 2). Multivariable analysis adjusting for infertility attributed to any reason (HR, 1.27; 95% CI, 1.09-1.48) instead of infertility attributed to ovulatory disorder was similar (shown in model 1 in eTable 1 in the Supplement). Additional multivariable analyses included not adjusting for OC use and duration (HR, 1.29; 95% CI, 1.11-1.49) (shown in model 2 in eTable 1 in the Supplement), adjusting for HT (HR, 1.17; 95% CI, 1.00-1.35) (shown in model 3 in eTable 1 in the Supplement), and adjusting for analgesic medication use (HR, 1.28; 95% CI, 1.10-1.48) (shown in model 4 in eTable 1 in the Supplement). None of the medications assessed were mediators of endometriosis and ENM. The proportion of endometriosis outcome that was mediated by analgesics was small, with a nonsignificant test for mediation (<1% to 1.1%; *P* = .12).

**Table 2.** Multivariable-Adjusted Associations of Laparoscopically Confirmed Endometriosis With Early Natural Menopause^a^

Laparoscopically confirmed endometriosis	Early natural menopause cases/person-years	Hazard ratio (95% CI)		
		Model 1	Model 2	Model 3
Without	2345/1 508 462	1 [Reference]	1 [Reference]	1 [Reference]
With	197/79 290	1.51 (1.30-1.74)	1.46 (1.26-1.69)	1.28 (1.10-1.48)

^a^ Each model is described in the Methods section.

We did not observe a difference in the association between endometriosis and ENM by BMI (<25 vs ≥25: HR, 1.20 [95% CI, 0.99-1.45] vs 1.43 [95% CI, 1.11-1.83; *P* for heterogeneity = .34), cigarette smoking status (never vs ever: HR, 1.36 [95% CI, 1.13-1.65] vs 1.11 [95% CI, 0.87-1.42]; *P* for heterogeneity = .57), or history of infertility attributed to ovulatory disorder (no vs yes: HR, 1.28 [95% CI, 1.08-1.51] vs 1.28 [95% CI, 0.90-1.82]; *P* for heterogeneity = .86) (Table 3). However, we did observe significant differences in the association between endometriosis and ENM by OC use (never vs ever: HR, 2.03 [95% CI, 1.34-3.06] vs 1.20 [95% CI, 1.02-1.42]; *P* for heterogeneity = .02) and nulliparity (nulliparous vs parous: HR, 1.46 [95% CI, 1.15-1.86] vs 1.14 [95% CI, 0.94-1.39]; *P* for heterogeneity = .05).

**Table 3.** Multivariable-Adjusted Associations of Laparoscopically Confirmed Endometriosis With ENM Stratified by Risk Factors of Age at Menopause

Laparoscopically confirmed endometriosis	ENM cases/person-years	HR (95% CI)		Model 2 *P* value for heterogeneity^c^
		Model 1^a^	Model 2^b^	
Stratified by BMI				
<25				.34
Without endometriosis	1464/862 052	1 [Reference]	1 [Reference]	
With endometriosis	124/47 683	1.43 (1.19-1.72)	1.20 (0.99-1.45)	
≥25				
Without endometriosis	871/642 496	1 [Reference]	1 [Reference]	
With endometriosis	73/31 378	1.61 (1.27-2.05)	1.43 (1.11-1.83)	
Stratified by cigarette smoking status				
Never				.57
Without endometriosis	1382/1 012 326	1 [Reference]	1 [Reference]	
With endometriosis	123/53 085	1.58 (1.31-1.90)	1.36 (1.13-1.65)	
Ever				
Without endometriosis	952/486 625	1 [Reference]	1 [Reference]	
With endometriosis	72/25 848	1.33 (1.05-1.70)	1.11 (0.87-1.42)	
Stratified by OC use				
Never				.02
Without endometriosis	324/224 204	1 [Reference]	1 [Reference]	
With endometriosis	30/7793	2.54 (1.72-3.74)	2.03 (1.34-3.06)	
Ever				
Without endometriosis	1995/1 218 905	1 [Reference]	1 [Reference]	
With endometriosis	167/68 883	1.38 (1.18-1.62)	1.20 (1.02-1.42)	
Stratified by parity				
Nulliparous				.05
Without endometriosis	539/319 045	1 [Reference]	1 [Reference]	
With endometriosis	87/26 328	1.62 (1.29-2.04)	1.46 (1.15-1.86)	
Parous				
Without endometriosis	1805/1 189 158	1 [Reference]	1 [Reference]	
With endometriosis	110/52 957	1.31 (1.08-1.59)	1.14 (0.94-1.39)	
Stratified by history of infertility attributed to ovulatory disorder				
No				.86
Without endometriosis	2188/1 424 640	1 [Reference]	1 [Reference]	
With endometriosis	147/63 004	1.42 (1.20-1.68)	1.28 (1.08-1.51)	
Yes				
Without endometriosis	157/83 822	1 [Reference]	1 [Reference]	
With endometriosis	50/16 285	1.60 (1.15-2.22)	1.28 (0.90-1.82)	

Abbreviations: BMI, body mass index (calculated as weight in kilograms divided by height in meters squared); ENM, early natural menopause; HR, hazard ratio; OC, oral contraceptive.

^a^ Model 1 was adjusted for age and calendar time.

^b^ Model 2 included model 1 and was additionally adjusted for race and ethnicity (non-Hispanic White or other), BMI at 18 years (<18.5, 18.5 to <22.5, 22.5 to <25, or ≥25), current BMI (<18.5, 18.5 to <22.5, 22.5 to <25, 25 to <30, or ≥30), cigarette smoking status (never, past, or current), cigarette smoking pack-years (0, 1-5, 6-10, 11-15, 16-20, or 21-98), alcohol use (0, 0.1 to <10.0, 10 to 30, or ≥30 g/d), physical activity (<3, 3 to 8, 9 to 17, 18 to 26, or ≥27 metabolic equivalent task h/wk), vegetable protein (quintiles), estimated plasma 25-hydroxyvitamin D score (nanograms per milliliter; quintiles), age at menarche (≤11, 12-13, or ≥14 years), menstrual cycle length at age 18 to 22 years (<26, 26-31, or ≥32 days), OC use (never, past, or current), duration of OC use (0, 1-23, 24-47, 48-71, 72-95, 96-119, or ≥120 months), parity (nulliparous or parous: 1, 2, 3, or ≥4 children), total lactation duration (0 to <1, 1-6, >6-12, or ≥12 months), and history of infertility attributed to ovulatory disorder (no or yes).

^c^ *P* value test for heterogeneity compared the association of endometriosis with early natural menopause across potential effect modifier strata using the likelihood ratio test.

Results from sensitivity analyses that censored follow-up at the first report of HT were similar to the main findings (HR, 1.33; 95% CI, 1.11-1.60) (shown in model 3 in eTable 2 in the Supplement). However, when censoring at the first OC use, the risk of ENM was elevated compared with the main results (HR, 2.03; 95% CI, 1.34-3.06) (shown in model 3 in eTable 2 in the Supplement).

## Discussion

In this large, prospective cohort study, we observed that surgically confirmed endometriosis was associated with a significantly greater risk of ENM. The risk estimate remained robust and statistically significant with adjustment for demographic and behavioral factors. The association was slightly attenuated with additional adjustment for reproductive factors but remained statistically significant. Residual confounding may remain; however, for residual confounding to alter the conclusions that were drawn from the quantified effect estimates, it would have to exceed the confounding outcomes that were captured by the data that were already included in the multivariable-adjusted models. The observed association between endometriosis and ENM varied between subgroups, with statistically significant effect modifiers identified. Those with endometriosis had the highest risk for ENM among women who never used OCs or were nulliparous.

To our knowledge, no previous study has prospectively investigated the association between endometriosis and ENM with time-varying covariates, time-to-event analyses, and early age at natural menopause (clinically defined as before age 45 years).^35^ A cross-sectional study^27^ reported that women with ENM (defined as menopause at ≤49 years, the cohort’s median age at natural menopause) were more likely to report an endometriosis diagnosis after adjustment for social class, pack-years of smoking, and OC use (odds ratio [OR], 2.49; 99% CI, 1.42-4.37). Similarly, Yasui et al^36^ reported a history of endometriosis to be significantly associated with younger age at onset of menopause (median age at menopause onset, 50 years) after adjustment for age, age at menarche, number of pregnancies, current BMI, history of infertility, and ever smoking before menopause (OR, 1.33; 95% CI, 1.05-1.68). Although these findings are similar to the results of the current study, the study designs are different (cross-sectional with recalled age at menopause vs prospective cohort study), and the outcomes are defined differently (age at menopause in years vs dichotomized menopause before age 45 years). Unlike Yasui et al,^36^ we did not find that the association between endometriosis and ENM differed by history of infertility (attributed to ovulatory disorder). We believe that the present study expanded on these 2 previous investigations by using prospective data; applying a standard definition of ENM; and accounting for time-varying confounding by additional behavioral factors, such as physical activity, and by reproductive factors, such as menstrual cycle characteristics, parity, lactation, and duration of OC use, with the Cox proportional hazards regression model.

In addition, we examined the association between endometriosis and ENM after stratifying by BMI (<25 or ≥25). Previous studies found a consistent inverse association between overweight or obesity and risk of endometriosis.^4,23,37^ In the population of the present study, those with endometriosis were less likely to be overweight or obese at cohort baseline in 1989 and in late adolescence. Previous studies reported that women with a BMI between 25.0 and 29.9 had significant lower odds for ENM.^38^ We found that, among participants with a BMI of 25 or higher, endometriosis was associated with an approximately 60% greater risk for ENM. This finding suggests that, among women with a BMI of 25 or higher who may have lower risk for ENM, endometriosis emerges as a risk factor.

Endometriosis was associated with a greater risk for ENM among nulliparous vs parous participants. Parity has been associated with delayed age at menopause,^21^ and endometriosis was not a significant risk factor for ENM in this subgroup. Although a meta-analysis found an association between OC use and later age at natural menopause,^39^ a recent discovery in the Nurses’ Health Study II population^40^ did not support a clear association between duration of OC use (decreasing lifetime number of ovulatory cycles)^41^ and risk for ENM. In the present study, among participants who never used OCs, endometriosis was associated with a 2-fold greater risk for ENM. It is likely that OC use masks menopause, which is important to consider in this analysis particularly because women may use OCs to control endometriosis-associated symptoms. Similarly, women may use analgesics for endometriosis-associated symptoms; however, none of the analgesics we assessed were significant mediators of the association between endometriosis and ENM.

### Limitations

This study has several limitations. Censoring because of loss to follow-up or competing events (eg, surgical menopause) can represent potential selection bias in prospective studies. In the Nurses’ Health Study II cohort, both dropout and incidence of competing events were low; simulation studies have shown that, when overall censoring is low, the likelihood of potential bias in cohort studies is also low.^42,43^ Although we relied on self-report of endometriosis, which might lead to some exposure misclassification, the endometriosis data were collected prospectively before the outcome and therefore could not be differentially misclassified with respect to the outcome. Furthermore, self-report of endometriosis has been demonstrated to be highly valid in this Nurses’ Health Study II cohort of medical professionals.^29^ We also relied on self-report for onset of menopause, which might contribute to some outcome misclassification^44,45^; however, menopause status also was collected prospectively, repeated across multiple questionnaires, confirmed for reported status consistency, and unlikely to be reported more or less accurately by women with endometriosis compared with those without endometriosis. Self-assessment of menopause was validated in the Nurses’ Health Study I cohort, wherein age at menopause was confirmed with exceptional validity using medical records for 99% of participants, and 82% of participants consistently recalled the same age at menopause within 1 year on repeated questionnaires.^45^ To further account for potential masking of ENM among participants who were exposed to hormones, we conducted analyses that censored for HT, and the results did not change. The race and ethnicity of the study population were fairly homogenous, yet we would expect that the physiological association between the reproductive factors of endometriosis and ENM would not differ substantially by race and ethnicity. However, confirmation of these results in more diverse populations will be informative. This analysis included more than 100 000 women who contributed more than 1.5 million person-years of follow-up, during which 2542 ENM cases were reported. Nevertheless, low incidence of early menopause presented some limitations with estimating the risk among endometriosis-exposed (n = 6640) compared with unexposed (n = 99 993) participants, highlighting the challenge for longitudinal studies of uncommon outcomes and the need for large sample sizes and long follow-up duration.

## Conclusions

This prospective cohort study found a statistically significant association between laparoscopically confirmed endometriosis and risk for ENM. Endometriosis and ENM had many overlapping risk factors, suggesting that an association could be explained by confounding. After time-varying multivariable adjustment, little confounding by known demographic and behavioral risk factors for ENM was observed. There was some confounding impact after adjustment for reproductive factors. Endometriosis may be an important risk factor for ENM, and women with endometriosis, particularly those who are nulliparous and never-users of OCs, may be at a higher risk for a shortened reproductive duration.

## Notes

Supplement. **eTable 1.** Multivariable Adjusted Associations of Laparoscopically Confirmed Endometriosis With Early Natural Menopause, by Varying the Adjusted Variables (Infertility Due to Any Reason, Hormone Use, and Analgesic Use) **eTable 2.** Multivariable Adjusted Associations of Laparoscopically Confirmed Endometriosis With Early Natural Menopause, Additionally Censoring at First Report of Hormone Therapy, and Oral Contraceptive Use
