# Supplementary material for: Association Between Laparoscopically Confirmed Endometriosis and Risk of Early Natural Menopause
Source: JAMA Netw Open. 2022 Jan 21;5(1):e2144391. doi: 10.1001/jamanetworkopen.2021.44391 (PMC8783263; doi:10.1001/jamanetworkopen.2021.44391)
Supplement: Supplement. — eTable 1. Multivariable Adjusted Associations of Laparoscopically Confirmed Endometriosis With Early Natural Menopause, by Varying the Adjusted Variables (Infertility Due to Any Reason, Hormone Use, and Analgesic Use) eTable 2. Multivariable Adjusted Associations of Laparoscopically Confirmed Endometriosis With Early Natural Menopause, Additionally Censoring at First Report of Hormone Therapy, and Oral Contraceptive Use [file jamanetwopen-e2144391-s001.pdf]

Supplementary Online Content

Thombre Kulkarni M, Shafrir A, Farland LV, et al. Association between laparoscopically confirmed endometriosis and risk of early natural menopause. *JAMA Netw Open*. 2022;5(1):e2144391. doi:10.1001/jamanetworkopen.2021.44391

eTable 1. Multivariable Adjusted Associations of Laparoscopically Confirmed Endometriosis With Early Natural Menopause, by Varying the Adjusted Variables (Infertility Due to Any Reason, Hormone Use, and Analgesic Use)

eTable 2. Multivariable Adjusted Associations of Laparoscopically Confirmed Endometriosis With Early Natural Menopause, Additionally Censoring at First Report of Hormone Therapy, and Oral Contraceptive Use

This supplementary material has been provided by the authors to give readers additional information about their work.

© 2022 Thombre Kulkarni M et al. *JAMA Network Open.*

eTable 1. Multivariable Adjusted Associations of Laparoscopically Confirmed Endometriosis With Early Natural Menopause, by Varying the Adjusted Variables (Infertility Due to Any Reason, Hormone Use, and Analgesic Use)

**Endometriosis**	**Early Natural Menopause Cases/Person-years**	**Hazard Ratio (95% Confidence Interval)**			
		**Model 1**	**Model 2**	**Model 3**	**Model 4**
No	2345 / 1,508,462	1.0 (Referent)	1.0 (Referent)	1.0 (Referent)	1.0 (Referent)
Yes	197 / 79,290	1.27 (1.09-1.48)	1.29 (1.11-1.49)	1.17 (1.00-1.35)	1.28 (1.10-1.48)

Model 1: Adjusted for age, calendar time, race/ethnicity (non-Hispanic White, Other), BMI at age 18 years (<18.5, 18.5 to <22.5, 22.5 to <25, 25+ kilograms/meter^2), current BMI (<18.5, 18.5 to <22.4, 22.5 to <24.9, 25 to < 30, 30+ kilograms/meter^2), cigarette smoking (never, past, current), cigarette smoking pack years (0, 1 ≤5, 6≤10, 11≤15, 16 ≤20, 21-98), alcohol (0, 0.1-<10.0, 10 -30, >=30 g/day) physical activity (<3, 3-8.9, 9-17.9, 18-26.9, >=27 metabolic equivalent task h/wk), vegetable protein (quintiles), estimated plasma 25-hyroxyvitamin D score (nanograms/milliliter, quintiles), age at menarche (≤11, 12-13, ≥ 14 years), menstrual cycle length at age 18-22 years (<25, 26-31, 32+ days), oral contraceptive use (never, past, current), duration of oral contraceptive use (0, 1-23, 24-47, 48-71, 72-95, 96-119, 120+ months) parity (nulliparous, parous:-1, 2, 3 ,≥ 4), total lactation duration (0- <1 month, 1-6, >6-12, ≥12 months), infertility attributed to any reason (no, yes)

© 2022 Thombre Kulkarni M et al. *JAMA Network Open.*

Model 2 Not adjusted for OC use and duration: Adjusted for age, calendar time, race/ethnicity (non-Hispanic White, Other), BMI at age 18 years (<18.5, 18.5 to <22.5, 22.5 to <25, 25+ kilograms/meter^2), current BMI (<18.5, 18.5 to <22.5, 22.5 to <24.9, 25 to < 30, 30+ kilograms/meter^2), cigarette smoking (never, past, current), cigarette smoking pack years (0, 1≤5, 6≤10, 11≤15, 16 ≤20, 21-98), alcohol (0, 0.1-<10.0, 10 -30, >=30 gm/day) physical activity (<3, 3-8.9, 9-17.9, 18-26.9, >=27 metabolic equivalent task h/wk), vegetable protein (quintiles), estimated plasma 25-hydroxyvitamin D score (nanograms/milliliter, quintiles), age at menarche (≤11, 12-13, ≥ 14 years), menstrual cycle length at age 18-22 years (<25 , 26-31, 32+ days), parity (nulliparous, parous:- 1, 2, 3 ,≥ 4), total lactation duration (0<1 month, 1-6, >6-12, ≥12 months), infertility attributed to ovulatory disorder (no, yes)

Model 3: Adjusted for age, calendar time, race/ethnicity (non-Hispanic White, Other), BMI at age 18 years (<18.5, 18.5 to <22.5, 22.5 to <25, 25+ kilograms/meter^2), current BMI (<18.5, 18.5 to <22.4, 22.5 to <24.9, 25 to < 30, 30+ kilograms/meter^2), cigarette smoking (never, past, current), cigarette smoking pack years (0, 1≤5, 6≤10, 11≤15, 16 ≤20, 21-98), alcohol (0, 0.1-<10.0, 10 -30, >=30 gm/day) physical activity (<3, 3-8.9, 9-17.9, 18-26.9, >=27 metabolic equivalent-h/wk), vegetable protein (quintiles), estimated plasma 25-hydroxyvitamin D score (nanograms/milliliter, quintiles), age at menarche (≤11, 12-13, ≥ 14 years), menstrual cycle length at age 18-22 years (<25 , 26-31, 32+ days), oral contraceptive use (never, past, current), duration of oral contraceptive use (0, 1-23, 24-47, 48-71, 72-95,96-119, 120 months) parity (nulliparous, parous:-1, 2, 3 ,≥ 4), total lactation duration (0 <1 month, 1-6, >6-12, ≥12 months), infertility attributed to ovulatory disorder (no, yes), and hormone use (no, yes).

Model 4: Adjusted for age, calendar time, race/ethnicity (non-Hispanic White, Other), BMI at age 18 years (<18.5, 18.5 to <22.5, 22.5 to <25, 25+ kg/m^2), current BMI (<18.5, 18.5 to <22.4, 22.5 to <24.9, 25 to < 30, 30+ kilograms/meter^2),cigarette smoking (never, past, current), cigarette smoking pack years (0, 1 ≤5, 6≤10, 11≤15, 16 ≤20, 21-98), alcohol (0, 0.1-<10.0, 10 -30, >=30 g/day) physical activity (<3, 3-8.9, 9-17.9, 18-26.9, >=27 metabolic equivalent-h/wk), vegetable protein (quintiles), estimated plasma vitamin 25 hydroxyvitamin D score (nanograms/milliliter quintiles), age at menarche (≤11, 12-13, ≥ 14 years), menstrual cycle length at age 18-22 years (<25 , 26-31, 32+ days), oral contraceptive use (never, past, current), duration of oral contraceptive use (0, 1-23, 24-47, 48-71, 72-95,96-119, 120 months) parity (nulliparous, parous:-1, 2, 3 ,≥ 4), total lactation duration (0 <1 month, 1-6, >6-12, ≥120 months), infertility attributed to ovulatory disorder (no, yes), and analgesic use (yes/no). Analgesic variable was created from the following variables: aspirin, acetaminophen, ibuprofen, non-steroidal anti-inflammatory drugs, and Cox inhibitor

© 2022 Thombre Kulkarni M et al. *JAMA Network Open.*

eTable 2. Multivariable Adjusted Associations of Laparoscopically Confirmed Endometriosis With Early Natural Menopause, Additionally Censoring at First Report of Hormone Therapy, and Oral Contraceptive Use

Laparoscopically-Confirmed Endometriosis	ENM cases/person-years	HR (95% CI)		
		Model 1	Model 2	Model 3
**Censoring on first use of hormone replacement therapy**				
Without Endometriosis	1836 / 1 287 844	1.00 (Referent)	1.00 (Referent)	1.00 (Referent)
With Endometriosis	129 / 54 493	1.53 (1.28-1.83)	1.48 (1.24-1.78)	1.33 (1.11-1.60)
**Censoring on past and current oral contraceptive use**				
Without Endometriosis	324 / 224 204	1.00 (Referent)	1.00 (Referent)	1.00 (Referent)
With Endometriosis	30 / 7793	2.54 (1.72-3.74)	2.54 (1.72-3.76)	2.03 (1.34-3.06)

Abbreviations: ENM=Early Natural Menopause; HR = hazard ratio; CI = confidence interval

Model 1: Adjusted for age and calendar time

Model 2 included model 1 and was additionally adjusted for race and ethnicity (non-Hispanic White, Other), BMI at age 18 years (<18.5, 18.5 to <22.5, 22.5 to <25, 25+ kilograms/meter^2), current BMI (<18.5, 18.5 to <22.5, 22.5 to <24.9, 25 to < 30, 30+, kilograms/meter^2), cigarette smoking status (never, past, current), cigarette smoking pack years (0, 1≤5, 6≤10, 11≤15, 16 ≤20, 21-98), alcohol use (0, 0.1-<10.0, 10 -30, >=30 g/day) physical activity (<3, 3-8.9, 9-17.9, 18-26.9, >=27 metabolic equivalent task h/wk), vegetable protein (quintiles), estimated plasma 25 hydroxyvitamin D score nanograms/milliliter (quintiles)

Model 3 included model 2 and was additionally adjusted for age at menarche (≤11, 12-13, ≥ 14 years), menstrual cycle length at age 18-22 years (<25 , 26-31, 32+ days), parity (nulliparous, parous:-1, 2, 3 ,≥ 4), total lactation duration (0-<1 month, 1-6, >6-12, ≥12 months), history of infertility attributed to ovulatory disorder (no, yes)

© 2022 Thombre Kulkarni M et al. *JAMA Network Open.*
